# Retraction Statement: Exosomal miR‐1255b‐5p targets human telomerase reverse transcriptase in colorectal cancer cells to suppress epithelial‐to‐mesenchymal transition

**DOI:** 10.1002/1878-0261.13452

**Published:** 2023-05-25

## Abstract

The above article, published online on 17 July 2020 in Wiley Online Library (wileyonlinelibrary.com), has been retracted by agreement between the journal Editor in Chief, Kevin Ryan and John Wiley and Sons Ltd. The retraction has been agreed following an investigation into concerns raised by a third party, which revealed inappropriate duplications of image panels within the article (multiple panels of fig. 2G and 3C), as well as inappropriate duplications of panels in figs 1D, 2G, and 3C with another study [1], which shares two of the authors. Compelling raw data was not available. Thus, the editors consider the conclusions of this manuscript substantially compromised.

[1] Exosomal miR‐128‐3p Promotes Epithelial‐to‐Mesenchymal Transition in Colorectal Cancer Cells by Targeting FOXO4 via TGF‐β/SMAD and JAK/STAT3 Signaling DOI:10.3389/fcell.2021.568738. Front. Cell Dev. Biol., 09 February 2021.

Reference

1 Zhang X, Bai J, Yin H, Long L, Zheng Z, Wang Q, et al. Exosomal miR‐1255b‐5p targets human telomerase reverse transcriptase in colorectal cancer cells to suppress epithelial‐to‐mesenchymal transition. Mol Oncol. 2020;14:2589–608. https://doi.org/10.1002/1878-0261.12765

The above article, published online on 17 July 2020 in Wiley Online Library (wileyonlinelibrary.com), has been retracted by agreement between the journal Editor in Chief, Kevin Ryan and John Wiley and Sons Ltd. The retraction has been agreed following an investigation into concerns raised by a third party, which revealed inappropriate duplications of image panels within the article (multiple panels of fig. 2G and 3C), as well as inappropriate duplications of panels in figs 1D, 2G, and 3C with another study [1], which shares two of the authors. Compelling raw data was not available. Thus, the editors consider the conclusions of this manuscript substantially compromised.

[1] Exosomal miR‐128‐3p Promotes Epithelial‐to‐Mesenchymal Transition in Colorectal Cancer Cells by Targeting FOXO4 via TGF‐β/SMAD and JAK/STAT3 Signaling DOI:10.3389/fcell.2021.568738. Front. Cell Dev. Biol., 09 February 2021.

Reference

1 Zhang X, Bai J, Yin H, Long L, Zheng Z, Wang Q, et al. Exosomal miR‐1255b‐5p targets human telomerase reverse transcriptase in colorectal cancer cells to suppress epithelial‐to‐mesenchymal transition. Mol Oncol. 2020;14:2589–608. https://doi.org/10.1002/1878-0261.12765

